# Decoding Attempted Hand Movements in Stroke Patients Using Surface Electromyography

**DOI:** 10.3390/s20236763

**Published:** 2020-11-26

**Authors:** Mads Jochumsen, Imran Khan Niazi, Muhammad Zia ur Rehman, Imran Amjad, Muhammad Shafique, Syed Omer Gilani, Asim Waris

**Affiliations:** 1 Department of Health Science and Technology, Aalborg University, 9220 Aalborg Øst, Denmark; imrankn@hst.aau.dk; 2 Centre for Chiropractic Research, New Zealand College of Chiropractic, Auckland 1060, New Zealand; imran.amjad@nzchiro.co.nz; 3 Health and Rehabilitation Research Institute, AUT University, Auckland 1010, New Zealand; 4 Faculty of Rehabilitation and Allied Sciences & Faculty of Engineering and Applied Sciences, Riphah International University, Islamabad 44000, Pakistan; ziaur.rehman@riphah.edu.pk (M.Z.u.R.); muhammad.shafique@riphah.edu.pk (M.S.); 5 Department of Biomedical Engineering & Sciences, School of Mechanical & Manufacturing Engineering, National University of Sciences and Technology (NUST), Islamabad 44000, Pakistan; omer@smme.nust.edu.pk (S.O.G.); asim.waris@smme.nust.edu.pk (A.W.)

**Keywords:** stroke, EMG, brain-computer interface, myoelectric control, pattern recognition

## Abstract

Brain- and muscle-triggered exoskeletons have been proposed as a means for motor training after a stroke. With the possibility of performing different movement types with an exoskeleton, it is possible to introduce task variability in training. It is difficult to decode different movement types simultaneously from brain activity, but it may be possible from residual muscle activity that many patients have or quickly regain. This study investigates whether nine different motion classes of the hand and forearm could be decoded from forearm EMG in 15 stroke patients. This study also evaluates the test-retest reliability of a classical, but simple, classifier (linear discriminant analysis) and advanced, but more computationally intensive, classifiers (autoencoders and convolutional neural networks). Moreover, the association between the level of motor impairment and classification accuracy was tested. Three channels of surface EMG were recorded during the following motion classes: Hand Close, Hand Open, Wrist Extension, Wrist Flexion, Supination, Pronation, Lateral Grasp, Pinch Grasp, and Rest. Six repetitions of each motion class were performed on two different days. Hudgins time-domain features were extracted and classified using linear discriminant analysis and autoencoders, and raw EMG was classified with convolutional neural networks. On average, 79 ± 12% and 80 ± 12% (autoencoders) of the movements were correctly classified for days 1 and 2, respectively, with an intraclass correlation coefficient of 0.88. No association was found between the level of motor impairment and classification accuracy (Spearman correlation: 0.24). It was shown that nine motion classes could be decoded from residual EMG, with autoencoders being the best classification approach, and that the results were reliable across days; this may have implications for the development of EMG-controlled exoskeletons for training in the patient’s home.

## 1. Introduction

A stroke is a cardiovascular disease affecting millions of people each year, where approximately 80% of the survivors are left with motor disabilities, such as paresis or paralysis [1,2]. Even after rehabilitation, around 50% of the patients are left with disabilities such that they require assistance with some of their activities of daily living [3,4]. Since a stroke is heterogeneous, there is no effective treatment that works for all [2]. There seems to be a consensus that principles of motor learning are relevant to stroke recovery [5], and induction of neural plasticity, which is the underlying factor of motor learning [6]. New patient-driven technologies have emerged where motor learning principles, such as repetition and attention, are incorporated in training, examples of such technologies are muscle- and brain-triggered exoskeletons or robots [7,8,9,10]. With the advances in the design and production of exoskeletons and rehabilitation robots, it is possible to perform different motions, which can be used to introduce task variability in training that can maximize the retention and generalization of the relearned movements [5]. To use such devices, however, it is necessary to detect the movement intention of various movement types to pair the motor commands with relevant afferent feedback. In patients with paralysis and no detectable electromyography (EMG) activity, it is necessary to use a Brain-Computer Interface where movement intentions are detected through electroencephalography (EEG). It is possible to classify movement intentions from idle activity with accuracies up to 80% in stroke patients [11,12], but the accuracies decrease when different movement types are classified [11,12,13]. If EMG activity is preserved or regained [14], it is possible to classify various movement types with high accuracy [15], even in patients with severe impairments [16,17]. It has been shown that neuroplasticity can be introduced using both EEG- and EMG-triggered electrical stimulation for providing afferent feedback [18], but it may be advantageous to use EMG if different movement types need to be classified. It has been shown in different studies that different movement types can be classified from EMG activity from the muscles in the affected limb. These movements include finger movements [14], various functional hand movements, such as open/close [15,19,20,21,22] and grasps [23], wrist extension [16], elbow and shoulder movements [24], and reaching [25]. Some of the techniques that have been used for decoding the attempted movements from the EMG are amplitude thresholds of the EMG signal envelope and proportional control [14,20], and pattern recognition approaches using, e.g., Hudgins time-domain features [15], autoregressive coefficients [22], empirical mode decomposition [26], and wavelets [27]. The performance of the decoding algorithms spans a wide range of 38–100%. Generally, the highest accuracies were associated with binary tasks, such as detecting a movement versus no-movement, and the performance decreases when including more motion classes. Some results have also shown how the classification accuracy is affected by the severity of the stroke [21]. As outlined, several studies have investigated the possibility of decoding attempted movements from stroke patients using surface EMG. These studies have primarily been single-session studies; therefore, there is a need for reliability studies to see if the decoding results are reproducible over time. Moreover, in previous work, EMG electrodes have been positioned for each patient individually to account, e.g., for muscle weakness and spasticity [22], or several EMG electrodes have been used to capture the activity from several muscles [15]. Therefore, the aim of this study was to investigate if different hand and forearm movements can be classified using a simple electrode setup placed on the same three muscles across heterogeneous stroke participants over two different days using a simple pattern recognition (linear discriminant analysis) approach with a low computational complexity which implements low-cost embedded systems. For comparison purposes, the simple classification approach using linear discriminant analysis was compared to autoencoders and convolutional neural networks that have been shown previously to improve the classification performance [28], but are more computationally intensive. In addition, the reliability of the pattern recognition approach was evaluated over two days. Besides the reliability analysis, it was investigated if it was possible to use the EMG recorded on one day to classify the EMG on the other day. Lastly, it was investigated if there was an association between the level of motor impairment and classification accuracy. Such an association has been reported previously, but more evidence is important, especially in a heterogeneous condition, such as a stroke.

## 2. Materials and Methods

### 2.1. Participants

Sixteen stroke patients (one female; 53 ± 8 years old) were recruited for this study (see the patient demographics in Table 1) from Railway General Hospital in Rawalpindi, Pakistan. One patient dropped out during the data collection. All patients provided their informed consent prior to participation. The procedures were approved by the local ethical committee (Riphah/RCRS/REC/00651). All procedures were in accordance with the Declaration of Helsinki. The Fugl-Meyer Assessment was performed to indicate the motor impairment of the patients, (motor score). The motor part of the Fugl-Meyer Assessment consists of two scores (100 points in total), one for the upper (66 points) and lower extremities (34 points). In this study, the score for the upper extremities is of interest. It covers the functionality of shoulder, elbow, wrist, and finger movements, as well as grasping various objects [29].

### 2.2. Recordings—Surface EMG

Six surface EMG electrodes (Ambu Neuroline 720 surface electrodes, REF 72000-S/25, Ambu, Ballerup, Denmark) were placed on the forearm on Extensor Carpi Radialis, Flexor Carpi Radialis, and Flexor Carpi Ulnaris. Two electrodes were placed on each muscle two cm apart and used in a bipolar configuration to obtain a single channel. The signals were referenced to a moist wristband. The signals were amplified with a gain of 10,000 (OT Bioelettronica, Torino, Italy) and sampled with 2048 Hz.

### 2.3. Experimental Setup

The experiment consisted of two recording sessions performed on two different days. The same experimental procedure was followed in both sessions. The recordings were performed in a seated position. Initially, the EMG electrodes were placed on the forearm on the most affected side, and the signal quality was checked (the electrode positions were marked on the forearm to ensure the same placement of the electrodes on day two). The participants were instructed how to perform the motions, and during the recording of the signals, they were visually cued (a picture of the specific motion was shown). A digital trigger was sent to the amplifier to synchronize the visual cue with the EMG recordings at the beginning of the recording. The following motion classes were performed: Hand Close, Hand Open, Wrist Extension, Wrist Flexion, Supination, Pronation, Lateral Grasp, Pinch Grasp, and Rest. Each motion class consisted of six repetitions of attempted movement, and the participant was asked to maintain the contraction for six seconds. Between each movement, there was a break of six seconds. All repetitions of the motion class were completed before moving to the next motion class. The order of motion classes was randomized.

### 2.4. Data Analysis

#### 2.4.1. Pre-Processing and Feature Extraction

**Figure 1:**
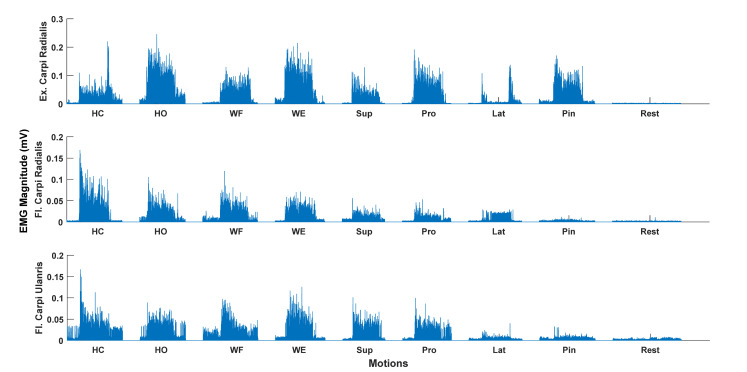
Rectified (only for visualization) and bandpass filtered surface EMG for the nine different motion classes for a single repetition and a single participant. Hand Close (HC), Hand Open (HO), Wrist Flexion (WF), Wrist Extension (WE), Supination (Sup), Pronation (Pro), Lateral Grasp (Lat), and Pin (Pinch Grasp). Flexor (Fl.), Extensor (Ex.). Clear EMG activity can be seen for most motion classes except the Lateral Grasp.

The EMG was bandpass filtered between 20–500 Hz, and a Notch filter from 48–52 Hz was applied using a 2nd order Butterworth filter with zero phase shift. The onsets of the EMG activity were visually inspected to avoid a potential delay between the cue and onset of the movements, such that the movement onsets were correctly identified for further analysis. Each of the 6-s repetitions of the motion class were extracted, and the first and last second were removed from the analysis, which resulted in epochs of 4-s duration for each repetition of the motion class. Following the pre-processing, four features were extracted: Mean absolute value, waveform length, zero crossing, and slope sign changes [30]. The features were extracted from a 200-millisecond data window with no overlap to obtain more data for classification [31]. The same analysis was performed on the data from the two separate recording sessions. An example of the filtered and rectified EMG for each motion class is shown in Figure 1.

#### 2.4.2. Classification

The classification was performed in two different ways: (1) Within-session calibration; and (2) between-session calibration. For the within-session calibration, 80% of the data windows were randomly selected for training, and 20% of the data windows were used for testing. The classifiers were trained on data windows from each subject individually and on the same randomly selected data windows to fairly compares classifiers. In the within-session calibration, the classifier was trained and tested on the recordings from the same day. In the between-session calibration, the classifier was trained on data from one day and tested on the other day. Moreover, confusion matrices were obtained. 

Three classifiers were tested, two of them used features as input, while the third used bandpass filtered data windows as input. The features were classified using a linear discriminant analysis classifier (LDA) and autoencoders (AE), and the filtered data windows were classified with a convolutional neural network (CNN). The LDA is a linear classifier that can separate multiple classes using a linear combination of the input features [32], while AE is an artificial neural network. In this study, the default MATLAB implementation of the LDA was used where all classes have the same covariance matrix. In the implementation of the LDA in this study, all motion classes were included leading to a classification problem with nine classes. The AE network consisted of two layers, each with hidden units of 12 (length of the feature vector) and a softmax layer. Optimized parameters from previous work were used [33]. The 200-millisecond data windows of EMG were classified using a CNN. The CNN consisted of an input layer (200-millisecond data window), four convolutional layers, each with Relu and pooling layers, a fully connected layer, and a softmax layer. The architecture of the network was optimized randomly, and the network was trained using Adam optimizer with default values except for L2R (10 × 10^−6^) and initial learning rate (5 × 10^−3^) with a ‘piecewise’ learning rate schedule having a drop rate factor of 0.1 and drop period of 4. Maxepochs were set to 20, and a mini-batch size of 16 and 32 were used for within- and between-session analyses, respectively. All data processing and analyses were performed in MATLAB 2020a (MathWorks^®^). The computational time of the different classifiers was estimated on the training and test data. The classification was performed on a computer with 8 GB RAM, a core i5 processor, and a 64-bit operating system.

### 2.5. Statistics

All statistical analyses were performed in IBM^®^ SPSS^®^. The test-retest reliability of the classification accuracies in the within- and between-session calibration was assessed using a two-way mixed-effect model with absolute agreement. The test was repeated three times for LDA, AE, and CNN, respectively. The mean classification accuracy was calculated across the two days for the within- and between-session calibration, and a two-way repeated-measures analysis of variance (ANOVA) was performed with “Calibration” (2 levels: Within-, and between-session calibration) and “Classifier” as factors (3 levels: LDA, AE, and CNN). Six Friedman tests were performed (the assumption of normality was violated) on the diagonal values in the confusion matrices (mean across the two days) with “Motion Class” as the factor (9 levels: Hand Close, Hand Open, Wrist Extension, Wrist Flexion, Supination, Pronation, Lateral Grasp, Pinch Grasp, and Rest) for the three classifiers in the within- and between-session calibration. Significant tests were followed up with a posthoc test using Bonferroni correction. Lastly, the Spearman correlation coefficient was calculated between the upper limb Fugl-Meyer score and the average classification accuracy for the within-session calibration (mean across the two days). Significant tests in all analyses were assumed when *p* < 0.05.

## 3. Results

**Figure 2:**
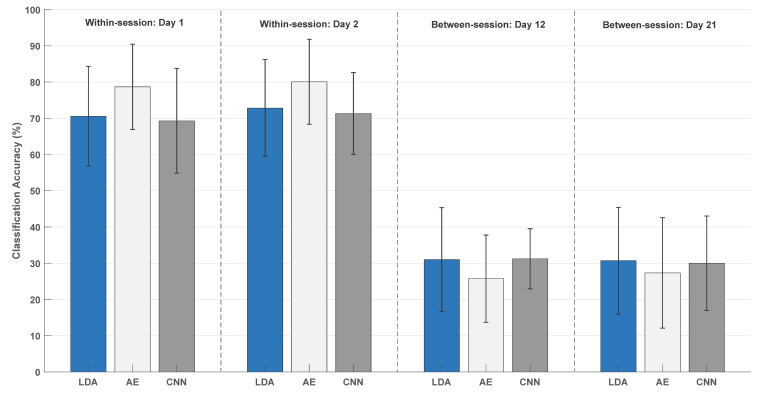
Overall classification accuracy for all motion types. The results are presented as mean ± standard deviation across participants. “Day12” indicates training on data from day 1 and testing on data from day 2. “Day21” indicates training on data from day 2 and testing on data from day 1. LDA (linear discriminant analysis), AE (autoencoders), and CNN (convolutional neural network).

The average classification accuracies across participants obtained in the within-session calibration were approximately 70% for the LDA and CNN on both days, while 80% of the motions were correctly classified with AE (see Figure 2). The average classification accuracies in the between-session calibration were approximately 30% for day 1 and 2, respectively, with slightly lower accuracies for AE. The results of the test-retest analysis are presented in Table 2. Good agreement was obtained for all classification and calibration scenarios except for the CNN in the between-session calibration, where the moderate agreement was obtained [34]. 

The mean classification accuracy was calculated across the two days, and a two-way repeated-measures ANOVA revealed a significant interaction between Calibration and Classifier (F_(2,28)_ = 27.05; *p* < 0.001; η^2^ = 0.66). This was followed up with two one-way repeated-measures ANOVA tests for the within- and between-session calibration. For the within-session calibration, there was a significant difference between the classifiers (F_(1.2,17.1)_ = 17.27; *p* < 0.001; η^2^ = 0.55), and the posthoc analysis revealed higher classification accuracies for AE compared to the LDA and CNN. For the between-session calibration, there was no difference between the classifiers (F_(2,28)_ = 3.10; *p* = 0.061; η^2^ = 0.18). 

The confusion matrices (mean across the two days and across participants) for the within-session and between-session calibration are shown in Table 3, Table 4, Table 5, Table 6, Table 7 and Table 8, respectively. For the within-session calibration, the highest numbers were on the diagonal for all motion classes, with Rest being the easiest to discriminate (92–95%). The other motion classes were in the range of 55–83%. A significant difference between the motion classes was found for the LDA (χ^2^_(8)_ = 44.13; *p* < 0.001), AE (χ^2^_(8)_ = 39.63; *p* < 0.001) and CNN (χ^2^_(8)_ = 51.14; *p* < 0.001). For the LDA, the posthoc analyses revealed that Rest had higher classification accuracies compared to the other classes except Wrist Extension and Hand Open. Wrist Extension and Hand Open had higher classification accuracies than Lateral Grasp. For AE, the classification accuracies for the Rest class were higher than Lateral Grasp, Supination, Pinch Grasp, and Pronation. For CNN, Rest had higher classification accuracies compared to the other classes except Wrist Extension and Wrist Flexion. Wrist Extension and Wrist Flexion had higher classification accuracies than Lateral Grasp.

**Figure 3:**
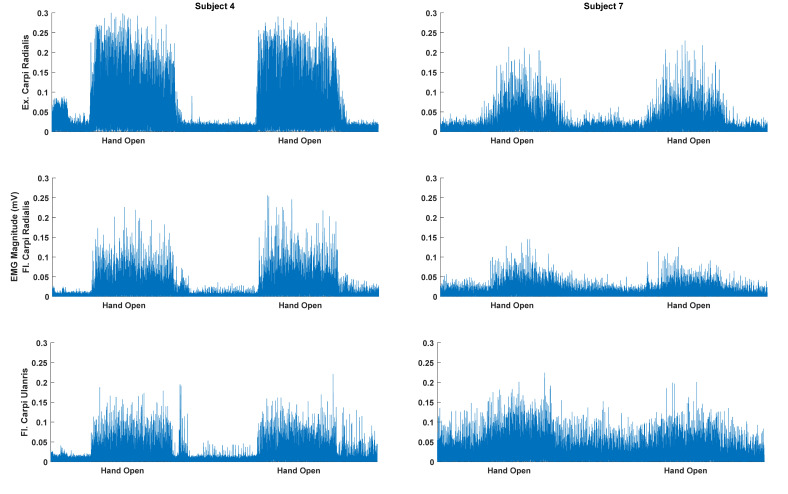
Rectified (only for visualization) and bandpass filtered surface EMG for the Hand Open motion class for the subject with the highest (subject 4) and lowest (subject 7) classification accuracy. The highest and lowest overall classification accuracies were 91% and 54% (classified with linear discriminant analysis), respectively. The amplitude of the EMG for the motions performed by the best subject is higher compared to the worst subject. Moreover, there is a smaller EMG amplitude for the resting state between the movements for the best subject.

For the between-session calibration, the highest numbers were on the diagonal for most motion classes except Lateral and Pinch Grasps. Rest was the motion class with the highest accuracies (22–66%). The other motion classes were in the range of 13–43%. A significant difference between the motion classes was found for the LDA (χ^2^_(8)_ = 23.02; *p* = 0.003), AE (χ^2^_(8)_ = 20.52; *p* = 0.009), and CNN (χ^2^_(8)_ = 29.72; *p* < 0.001). For the LDA, the posthoc analyses revealed no difference between the classes, which is due to the conservative nature of the Bonferroni correction. For AE, the classification accuracies for the Wrist Extension and Wrist Flexion classes were higher than the Pinch Grasp. For CNN, Rest had higher classification accuracies compared to the Lateral Grasp and Pinch Grasp. Wrist Extension had higher classification accuracies than the Pinch Grasp. There was a considerable standard deviation across the participants (see Figure 2), which may be attributed to the amplitude differences between movement and Rest for the different participants (see Figure 3).

The Spearman correlation was calculated between the upper limb Fugl-Meyer score and the classification accuracy across all motion classes for the within-session calibration. The results are presented in Table 9. There was no association between the functional score and the classification accuracies.

The results of the computational time are presented in Table 10. The LDA was the fastest classifier to train, followed by the AE. For testing, the LDA and AE were faster than the CNN, but it only took 0.22 and 0.27 s to classify all test data with a CNN.

## 4. Discussion

The aim of this study was to decode attempted movements in stroke patients. Generally, it was possible to decode nine different motion classes of the hand/forearm with accuracies of 79 ± 12% and 80 ± 12% for day 1 and 2, respectively (using AE). There was a strong intraclass correlation between the classification accuracies, but there was no association between the classification accuracies and the upper limb Fugl-Meyer score. The classification accuracies obtained with AE were significantly higher than the LDA and CNN in the within-session calibration, but not significantly different for between-session calibration.

The findings in this study are in agreement with other studies that have found that EMG of attempted movements can be decoded from stroke patients with motor impairments [15,16,17,21,22,24]. Similar or slightly lower classification accuracies are obtained, although the studies differ in terms of methodology. The number of channels in this study (three bipolar channels) is low compared to other studies [15,26], which is likely to account for some of the differences in the classification accuracies. Moreover, a generalized approach was used where the electrodes were placed on the same three muscles on the contrary to other findings where electrodes have been positioned based on the impairment of the individual patient [22]. By increasing the number of channels, it is possible to record activity from more muscles; especially the chronic stroke patients may have developed coping strategies to perform the movements using altered activation patterns. For the different grasp motions, it would have been beneficial to place EMG electrodes closer to or on the hand. Different classifiers were tested, and it was shown that the classification performance could be significantly improved using AE, which is in agreement with previous findings [28]. It was also shown that classification accuracies comparable to a LDA can be obtained for a CNN without the need for extracting features, but this indicates that it may not be needed to use a CNN over LDA with features. Generally, Hudgins time-domain features [15,22,24,27,30] have been used, but to improve the classification accuracies further, other feature types could be added to the feature vector, such as spectral information, wavelets, autoregressive coefficients, and entropy [15,27,35]. 

In future studies, the most optimal or general electrode setup and feature types could be investigated, e.g., with and without constraints on computational power and energy consumption of the system. For a low-end implementation, Raspberry Pi could be used, while a Parallella Baseline System (PBS) could be used when more computational power is needed with a constraint of still being energy efficient [36]. These approaches should be validated using online control and with afferent feedback from an exoskeleton. Lastly, the impairment of the patients would probably affect the classification accuracies as well. There was no association between the level of motor impairment and classification accuracy in this study, although that was expected, as it has been shown previously that classification accuracies decrease as a function of the severity of the injury [21]. The limited sample size in this study could explain that no association was found between the classification accuracies and motor impairment. Another explanation could be that the machine learning approach was able to pick up movement patterns from participants with a low Fugl-Meyer score leading to reasonably high classification accuracy, or that the Fugl-Meyer score not only reflects the tasks performed in this study, but also reflexes and shoulder and elbow movements. Participants that have reduced hand and wrist movement may have functional reflexes and elbow and shoulder movements. Lastly, the movements and assessment method in this study differed from those performed in Reference [21]. In Reference [21], more functional hand movement tasks were performed, while more wrist movement tasks where performed in the current study, which may be easier to perform for participants with a low Fugl-Meyer score compared to functional hand movement tasks leading to higher classification accuracy, and hence, a lower correlation coefficient. In addition, in Reference [21], they used the Stage of Hand component of the Chedoke-McMaster Stroke Assessment scale, which may be more sensitive to hand movements compared to the total upper limb Fugl-Meyer score. However, these are speculations that need to be tested in a future study.

The motion class that was easiest to discriminate was the Rest condition, which was significantly different from the other classes except Wrist Extension and Wrist Flexion in the within-session calibration. This indicates that the patients did not suffer much from spasticity, which would reduce the ability to discriminate between the motion classes and the usability of EMG to control an exoskeleton/rehabilitation robot or functional electrical stimulation. However, a recent study has found that spasticity can be reduced by utilizing a myoelectric computer interface [37]. The motion class that was most difficult to discriminate was the Lateral Grasp. This is probably because it resembles the motion class Hand Close, 7–13% of the data from the Lateral Grasp motion class was classified as Hand Close. It has also been previously reported that the motions that resembled each other were more difficult to classify [21]. In a rehabilitation scenario, the motion classes that are difficult to classify could be performed in separate training sessions to improve the system performance. The test-retest reliability of the classification of the motion classes was good for both within- and between-session calibration, but the 95%-percent confidence intervals were wide. In addition, there was a large standard deviation of the accuracies for the different participants. This could potentially be explained by various factors, such as the patient’s level of fatigue or that, the recruitment patterns of some motion classes differ slightly, and that the muscles from which the EMG was recorded were affected differently in the participants. Moreover, the amplitude of the EMG is related to the force the muscles can produce (see Figure 3). The classification accuracies may also be affected by the signal quality, which could be reduced over time if the impedance of the electrodes changed, due to, for example, sweating. The classification accuracies associated with the between-session calibration were significantly lower compared to the within-session calibration. However, if more days were included, it could potentially have improved the performance [28], but only 2–3 repetitions of each motion class are needed to perform within-session calibration [31], so it would be possible to quickly calibrate the EMG decoder each day. Another aspect that needs to be considered if an EMG-controlled exoskeleton/rehabilitation robot can be used by the patients in their own homes is if they can place the recording electrodes accurately on the muscles. An alternative is to use technology, such as the Thalmic Myo armband, which is easy to don and doff. It utilizes several dry electrodes around the forearm, and the activity from multiple muscles can be recorded. Previously it has been shown that comparable classification accuracies can be obtained using such a setup compared to state-of-the-art wet electrodes and expensive amplifiers [38].

## 5. Conclusions

In conclusion, it is possible to decode various motion classes of the hand and forearm in stroke patients using both a simple setup with few electrodes and a simple pattern recognition approach and a deep learning approach with and without feature extraction. These findings were consistent across days where the test-retest reliability was good. No association was found between the classification accuracies and the level of impairment. The Rest, Hand Open and Close, and Wrist Extension and Flexion were the classes that were easiest to classify, and the Lateral Grasp was the most difficult to classify. The best classification was obtained using AE. However, more patients with varying degrees of impairment should be included in future studies to validate these findings. Moreover, other feature types and electrode setups (number and location) should be investigated as well to improve the classification accuracy further. These approaches should be validated in online studies where afferent feedback is provided from an exoskeleton or rehabilitation robot.

## Figures and Tables

**Table 1:** Patient demographics. Upper limb (UL), and lower limb (LL). The maximum score is 66 and 34 for UL and LL, respectively.

Patient	Months Since Injury	Affected Side	Type of Injury	Fugl-Meyer [UL/LL/Total]
1	24	Left	Ischemic	[55/22/77]
2	17	Right	Ischemic	[36/34/70]
3	18	Right	Ischemic	[23/28/51]
4	32	Left	Ischemic	[46/32/78]
5	36	Left	Ischemic	[26/18/44]
6	5	Right	Ischemic	[65/31/96]
7	38	Right	Ischemic	[17/22/39]
8	2	Left	Ischemic	[59/31/90]
9	38	Right	Ischemic	[55/30/85]
10	6	Left	Ischemic	[51/23/74]
11	3	Right	Ischemic	[56/24/80]
12	5	Left	Hemorrhagic	[44/20/64]
13	66	Right	Hemorrhagic	[28/18/46]
14	19	Left	Ischemic	[50/21/71]
15	70	Left	Hemorrhagic	[36/33/69]

**Table 2:** Intraclass correlation coefficients for the different calibration scenarios for the three classifiers. The intraclass correlation coefficient and 95% confidence intervals are reported.

	Within-Session	Between-Session
Linear discriminant analysis	0.84 [0.54:0.95]	0.88 [0.63:0.96]
Autoencoders	0.88 [0.63:96]	0.87 [0.62:0.96]
Convolutional neural network	0.86 [0.58:0.95]	0.69 [0.06:0.90]

**Table 3:** Confusion matrix based on within-session calibration (the mean across the two days have been calculated) using linear discriminant analysis. All values are in percent and presented as the mean across participants. HC (Hand Close), HO (Hand Open), WE (Wrist Extension), WF (Wrist Flexion), Sup (Supination), Pro (Pronation), Lat (Lateral Grasp), and Pin (Pinch Grasp).

	HC	HO	WF	WE	Sup	Pro	Lat	Pin	Rest
HC	72	5	2	2	4	2	10	4	2
HO	4	77	6	4	2	3	1	4	1
WF	4	9	71	4	3	4	4	3	1
WE	2	4	4	75	6	3	2	5	1
Sup	2	2	2	7	66	8	7	5	3
Pro	1	2	2	2	12	70	4	7	3
Lat	9	2	1	2	9	8	56	9	6
Pin	2	4	1	5	7	3	6	69	5
Rest	0	1	0	0	2	2	2	3	92

**Table 4:** Confusion matrix based on within-session calibration (the mean across the two days have been calculated) using autoencoders. All values are in percent and presented as the mean across participants. HC (Hand Close), HO (Hand Open), WE (Wrist Extension), WF (Wrist Flexion), Sup (Supination), Pro (Pronation), Lat (Lateral Grasp), and Pin (Pinch Grasp).

	HC	HO	WF	WE	Sup	Pro	Lat	Pin	Rest
HC	82	3	2	2	2	1	8	3	1
HO	3	83	5	2	2	2	2	2	0
WF	2	7	80	3	2	2	3	3	1
WE	1	4	4	79	5	2	2	3	0
Sup	2	2	2	7	73	7	6	3	2
Pro	1	2	2	3	9	76	3	6	2
Lat	7	1	4	2	4	6	70	7	2
Pin	2	2	2	3	4	3	6	77	3
Rest	0	0	1	0	1	1	2	2	94

**Table 5:** Confusion matrix based on within-session calibration (the mean across the two days have been calculated) using a CNN. All values are in percent and presented as the mean across participants. HC (Hand Close), HO (Hand Open), WE (Wrist Extension), WF (Wrist Flexion), Sup (Supination), Pro (Pronation), Lat (Lateral Grasp), and Pin (Pinch Grasp).

	HC	HO	WF	WE	Sup	Pro	Lat	Pin	Rest
HC	70	5	4	2	3	1	13	3	1
HO	5	69	8	4	5	4	2	3	0
WF	2	8	73	6	3	4	3	3	0
WE	1	4	4	76	4	4	4	5	1
Sup	2	4	3	7	61	9	6	8	2
Pro	1	3	3	4	11	68	4	7	2
Lat	13	3	4	2	6	6	55	10	4
Pin	2	4	2	5	7	7	6	68	2
Rest	0	0	0	0	0	2	2	2	95

**Table 6:** Confusion matrix based on between-session calibration (the mean across the two days have been calculated) using linear discriminant analysis. All values are in percent and presented as the mean across participants. HC (Hand Close), HO (Hand Open), WE (Wrist Extension), WF (Wrist Flexion), Sup (Supination), Pro (Pronation), Lat (Lateral Grasp), and Pin (Pinch Grasp).

	HC	HO	WF	WE	Sup	Pro	Lat	Pin	Rest
HC	41	6	5	6	13	6	18	5	1
HO	16	30	13	6	14	7	9	7	1
WF	16	9	39	6	12	6	10	3	2
WE	15	7	6	42	7	3	10	10	2
Sup	17	9	10	4	21	10	17	11	3
Pro	12	6	7	4	15	23	12	15	7
Lat	35	8	4	5	14	7	17	6	5
Pin	19	8	7	6	15	12	6	24	6
Rest	13	0	4	1	14	9	5	11	43

**Table 7:** Confusion matrix based on between-session calibration (the mean across the two days have been calculated) using autoencoders. All values are in percent and presented as the mean across participants. HC (Hand Close), HO (Hand Open), WE (Wrist Extension), WF (Wrist Flexion), Sup (Supination), Pro (Pronation), Lat (Lateral Grasp), and Pin (Pinch Grasp).

	HC	HO	WF	WE	Sup	Pro	Lat	Pin	Rest
HC	29	9	16	6	8	7	22	6	1
HO	9	28	15	10	12	6	12	9	0
WF	16	9	40	8	13	7	6	3	1
WE	11	7	8	41	7	8	11	9	0
Sup	14	9	12	8	22	12	21	3	2
Pro	12	10	11	8	13	28	9	7	4
Lat	24	10	14	9	11	9	16	7	2
Pin	17	8	13	15	12	13	7	14	4
Rest	13	2	7	10	13	13	14	8	22

**Table 8:** Confusion matrix based on between-session calibration (the mean across the two days have been calculated) using a CNN. All values are in percent and presented as the mean across participants. HC (Hand Close), HO (Hand Open), WE (Wrist Extension), WF (Wrist Flexion), Sup (Supination), Pro (Pronation), Lat (Lateral Grasp), and Pin (Pinch Grasp).

	HC	HO	WF	WE	Sup	Pro	Lat	Pin	Rest
HC	30	8	19	5	14	4	14	7	1
HO	16	21	17	8	9	8	13	7	1
WF	8	11	49	7	10	4	8	3	2
WE	8	10	13	43	8	2	8	9	1
Sup	13	12	13	7	22	11	11	6	7
Pro	7	10	12	7	15	19	12	14	7
Lat	19	11	17	5	9	9	15	10	7
Pin	10	10	11	15	15	12	8	13	6
Rest	1	0	7	0	12	4	7	4	66

**Table 9:** Correlation analysis between the classification accuracies (mean across days) for the within-session calibration and the functional score (upper limb Fugl-Meyer score).

	Correlation Coefficients	*p*-Value
Linear discriminant analysis	0.29	0.30
Autoencoders	0.24	0.38
Convolutional neural network	0.37	0.18

**Table 10:** The computational time of the training and test data for within- and between-session calibration. In the within-session scenario, the training data consisted of 828 data windows, and the test data consisted of 198 test windows. For the between-session scenario, the training and test data consisted of 1026 data windows.

Classifier	Training (Seconds)	Test (Seconds)
Linear discriminant analysis (within-session)	0.010	0.010
Autoencoders (within-session)	12.16	0.015
Convolutional neural network (within-session)	47.68	0.22
Linear discriminant analysis (between-session)	0.018	0.018
Autoencoders (between-session)	13.22	0.016
Convolutional neural network (between-session)	58.77	0.27
